# Chronic health effects of recurrent indoor Pyrethroid exposure in enclosed workplaces: an occupational exposome–based clinical review in the context of limited safety guidance

**DOI:** 10.3389/fpubh.2026.1827623

**Published:** 2026-05-04

**Authors:** Ahmed Adel Mansour Kamar, Ioannis Mavroudis, Alin Ciobica, Diana Gheban, Irina-Luciana Gurzu

**Affiliations:** 1 Gulf of Suez Petroleum Company (GUPCO), Medical Department, Cairo, Egypt; 2 Clinical Recovery Hospital (Recuperare), UMF, Department of Orthopedics and Traumatology, Iasi, Romania; 3 Doctoral School, Alexandru Ioan Cuza University, Department Biology, Faculty of Biology, Iasi, Romania; 4 Neurosciences Department, Leeds Teaching Hospitals NHS Trust, Leeds, United Kingdom; 5 Laboratory of Neuropathology & Electron Microscopy, School of Medicine, Faculty of Health Sciences, Aristotle University of Thessaloniki (AUTH), Thessaloniki, Greece; 6 Alexandru Ioan Cuza University (UAIC), Department Biology, Faculty of Biology, Iasi, Romania; 7 “Ioan Haulica” Institute, Apollonia University, Iasi, Romania; 8 “Olga Necrasov” Center, Biomedical Research Group, Romanian Academy, Iasi, Romania; 9 CENEMED Platform for Interdisciplinary Research, University of Medicine and Pharmacy “Grigore T. Popa”, Iasi, Romania; 10 Faculty of Medicine, Grigore T. Popa University of Medicine and Pharmacy, Iasi, Romania

**Keywords:** chronic fatigue, HVAC, indoor pesticide exposure, liver enzymes, Occupational Exposure, pyrethroids, residue persistence, thyroid dysfunction

## Abstract

Pyrethroid insecticides are widely used indoors in residential, commercial, and occupational pest-control settings and are generally regarded as safe because of their relatively low acute toxicity. This perception is largely based on short-term exposure data and application practices conducted under well-ventilated conditions. However, indoor pesticide application does not always occur in environments that allow for rapid residue clearance.

In enclosed workplaces—particularly those with centralized heating, ventilation, and air-conditioning systems, limited fresh-air exchange, and dust-retaining surfaces such as wall-to-wall carpeting—pyrethroid residues may persist beyond the initial application period. These residues may accumulate in indoor dust and textiles and undergo resuspension, potentially resulting in recurrent low-level exposure. In such settings, exposure may involve not only pest-control personnel but also other workers who are not considered pesticide-exposed and are not routinely monitored.

From an occupational health perspective, this article draws on published environmental, biomonitoring, and clinical literature to discuss how building characteristics, residue persistence, and ventilation conditions may represent overlooked contributors to indoor pyrethroid exposure. Reported findings include neurologic symptoms, endocrine alterations—particularly involving thyroid function—and mild hepatic enzyme abnormalities, although evidence remains heterogeneous and non-causal. No exposure measurements or case investigations are presented. The aim of this review is to raise awareness and encourage further research on indoor exposure dynamics and workplace evaluation of indoor pesticide use.

The reviewed evidence indicates that indoor environments may sustain prolonged low-level exposure through reservoirs such as dust, carpets, and ventilation systems. Mechanistic data support potential multisystem involvement, while human studies suggest associations with neurological, endocrine, and hepatic changes, although findings remain variable and non-causal.

## 1 Introduction

Indoor insecticide use is common across residential, commercial, and occupational environments. Synthetic pyrethroids are frequently chosen because of their high insecticidal efficacy, broad availability, and perceived favorable safety profile compared with older organophosphorus pesticide classes (1–3). Consequently, indoor pyrethroid spraying has become a routine component of pest management programs in many buildings. While acute toxicity and neurotoxic manifestations at high doses are well described (4, 5), the potential health implications of chronic low-dose exposure in indoor settings remain less clearly addressed in current safety frameworks.

A central limitation of most indoor pesticide safety protocols is their focus on spray-time exposure and short-term re-entry precautions designed mainly to prevent acute intoxication (6, 7). These procedures typically emphasize evacuation during spraying, temporary ventilation, and avoidance of treated areas for a limited time. However, indoor environments differ fundamentally from outdoor application contexts because they contain multiple exposure reservoirs that can retain residues for long periods. Carpets, textiles, porous materials, and settled dust can accumulate pyrethroid residues and sustain low-level exposure long after application—especially when spraying is repeated and cleaning or ventilation is inconsistent (3, 8, 9). These protocols are primarily designed to prevent acute intoxication rather than address cumulative indoor exposure dynamics. In this review we particularly focus on repeated indoors spraying which often occurs without standardized clinical surveillance or clear spraying-frequency guidelines.

In enclosed buildings with central heating, ventilation, and air-conditioning (HVAC) systems, exposure dynamics are further influenced by airflow, recirculation, filtration efficiency, and redistribution of airborne particles. Even if air-conditioning is switched off during spraying, residues deposited on surfaces and dust reservoirs can later re-enter the breathing zone through resuspension, occupant movement, or ventilation restart. Wall-to-wall carpeting functions as an efficient sink for semi-volatile residues, enabling persistence within dust matrices and repeated re-aerosolization during routine activities such as walking or cleaning. Collectively, these processes support a chronic exposure paradigm that extends well beyond the initial spray event (3, 8, 10).

To conceptualize these complexes, time-extended exposure processes, an indoor pyrethroid exposure pathway framework (Exposome) provides a useful lens. This approach considers cumulative environmental exposures and associated biological responses over time, emphasizing that chronic disease risk is influenced by repeated low-dose exposures, chemical mixtures, and multiple exposure routes (11–13). Applying this concept, we define a recurrent indoor pyrethroid exposure pathway.

Mechanistically, Type II pyrethroids such as lambda-cyhalothrin act primarily by modulating voltage-gated sodium channels, leading to neuronal hyperexcitability (4, 5). However, growing evidence indicates that chronic low-dose exposure may also trigger broader biological pathways, including mitochondrial dysfunction, oxidative stress, and inflammatory signaling, which are relevant across neurological, endocrine, and cardiovascular systems (14–21). Oxidative stress, in particular, represents a cross-system mechanism linking prolonged exposure to neurotoxicity, endothelial dysfunction, and thyroid disruption. Because thyroid hormone synthesis is intrinsically oxidative, additional oxidative burden may plausibly influence thyroid homeostasis in susceptible individuals (17, 18, 21, 22).

Despite increasing recognition of indoor dust contamination with pyrethroids (2, 3, 8, 9), and environmental chemicals are known to affect neuro-endocrine and cardiometabolic systems (17–20, 22–24), an integrated clinical and occupational approach that integrates dust-reservoir persistence, resuspension-driven chronic exposure, and multisystem clinical outcomes and supporting biological pathways remains underdeveloped—especially in occupational settings characterized by enclosed, centrally air-conditioned, and carpeted environments. This potential gap may limit the effectiveness of current clinical recognition, surveillance, and exposure management, which may overlook residue-driven re-exposure loops, vulnerable populations, and building characteristics that influence exposure persistence and intensity.

Accordingly, this review integrates environmental, mechanistic, and clinical evidence on recurrent indoor pyrethroid exposure pathways that integrate residue persistence, dust and surface accumulation, and resuspension in enclosed workplaces, and their clinical relevance to thyroid, neurologic, and cardiovascular outcomes (23–25). Also, it may identify research priorities and occupational guidance needs for improved clinical surveillance and prevention, including residue monitoring, ventilation verification, and biomonitoring approaches to support early recognition, monitoring, and prevention of chronic health effects among potentially exposed workers.

### 1.1 Rationale and knowledge gap

Despite widespread use of pyrethroid insecticides indoors, existing safety frameworks address only acute exposure. Real-world conditions—such as repeated high-dose spraying in enclosed buildings with recirculated air and textile surfaces—illustrate the absence of evidence-based guidance on safe application, frequency, residue persistence, and chronic health effects. This clinical and occupational health guidance gap places workers and occupants at risk of long-term toxicity, underscoring the need to incorporate chronic exposure considerations into pesticide safety guidelines (23, 26, 27).

### 1.2 Research question

This review addresses how clinicians may recognize, evaluate, and monitor chronic health effects associated with recurrent indoor occupational pyrethroid exposure in enclosed workplaces, in the lack of evidence-based guidance on safe re-application frequency and ventilation requirements?

## 2 Methodology

This article is a clinical occupational medicine narrative review based on a structured examination of peer-reviewed biomedical and environmental literature. Relevant studies were identified through searches of PubMed/MEDLINE, Scopus, and Web of Science using keywords related to pyrethroids, indoor spraying, residue persistence, dust and surface contamination, HVAC systems, biomonitoring, and mechanistic health outcomes. Search terms included combinations of: “pyrethroids,” “indoor pesticide exposure,” “residue persistence,” “dust contamination,” “HVAC,” “biomonitoring,” “3-PBA,” “chronic exposure,” “thyroid,” “liver enzymes,” and “occupational exposure”. The literature search included studies published up to January 2026. Only English-language peer-reviewed studies were included. Titles and abstracts were initially screened for relevance, followed by full-text review of selected articles. Priority was given to publications addressing indoor residue persistence, resuspension processes, human biomonitoring or epidemiological associations, and mechanistic evidence relevant to neurological, endocrine, hepatic, cardiovascular, and immune effects. In total, approximately 120 articles were screened, of which around 40 key studies were included in the final narrative synthesis. Studies were selected based on relevance to indoor exposure scenarios, occupational settings, and reported human, environmental, or mechanistic evidence. Evidence was analyzed qualitatively to integrate environmental exposure dynamics with clinical and toxicological findings. A narrative approach was adopted to synthesize multidisciplinary findings in the absence of feasible quantitative analysis.

The included literature comprised environmental monitoring studies, human biomonitoring investigations, epidemiological analyses, and experimental mechanistic studies. Overall, findings consistently indicate the presence of indoor pyrethroid residues and recurrent low-level exposure, while human health outcomes remain heterogeneous and largely associative, with variability in study design, exposure assessment, and population characteristics.

## 3 Scientific background: pyrethroids, indoor use, and the rationale for an “indoor pyrethroid exposure pathway”

This section provides background scientific context to support interpretation of the reviewed evidence and is not derived directly from the literature synthesis.

Synthetic pyrethroids are the predominant class of insecticides used for indoor pest control due to their high insecticidal potency and comparatively low acute mammalian toxicity relative to older pesticide groups (1–4). Developed as photostable analogs of natural pyrethrins, these agents are widely applied in residential, commercial, and occupational environments. Commercial formulations vary in active ingredients and solvents, influencing volatility, residue behavior, and human exposure potential (3, 8).

Toxicologically, pyrethroids are categorized as Type I or Type II compounds depending on their chemical structure and neurotoxic profile. Type II agents—such as lambda-cyhalothrin, cypermethrin, and deltamethrin—contain an α-cyano group that prolongs the opening of voltage-gated sodium channels, leading to neuronal hyperexcitability and characteristic neurotoxic symptoms (4, 5). Although this mechanism explains acute toxicity, chronic low-dose exposure introduces additional biological stressors, including mitochondrial dysfunction, oxidative imbalance, and inflammatory signaling, that extend beyond classical neurotoxicity (14–16, 20, 21).

Indoor environments alter pesticide behavior compared with outdoor settings. Limited photodegradation and air exchange, combined with the hydrophobicity of pyrethroids, favor adsorption onto dust and textiles and long-term residue persistence (3, 8, 9). Carpets, curtains, and porous materials serve as efficient sinks, and in buildings with central HVAC systems, recirculated air can redistribute residue-laden dust throughout occupied zones (10, 28). These features enable chronic low-level re-exposure despite adherence to standard acute exposure controls.

The resuspension of dust particles during normal activities (e.g., walking, cleaning, or ventilation restart) is a key driver of ongoing exposure. This process allows residues to re-enter the breathing zone repeatedly, maintaining background exposure that may persist for weeks or months after spraying (8, 9). Such exposure loops are not addressed in standard risk-management frameworks that focus primarily on the spray event and immediate re-entry period (23, 26).

The exposure pathway framework describes how repeated applications, residue persistence, and resuspension create time-extended indoor exposure through inhalation, ingestion and contact routes (11–13). Also, it emphasizes that repeated, low-dose exposure can plausibly activate overlapping mechanistic pathways—oxidative stress, endocrine disruption, neuroimmune signaling, and vascular dysregulation—relevant to chronic multisystem toxicity (14–22).

Furthermore, chronic chemical exposures may contribute to building-related symptom complexes such as *Sick Building Syndrome (SBS)-like symptoms*, characterized by headaches, mucosal irritation, fatigue, and cognitive difficulty which improve upon leaving the environment (28, 29). Recurrent pesticide use indoors may represent an unrecognized component of SBS-like symptoms rather than a defined disease entity, sustained by residue reservoirs and inadequate ventilation. Recognizing this link reinforces the need for comprehensive indoor-air management and chronic-exposure assessment rather than reliance on acute toxicity models. Sick Building Syndrome is multifactorial and cannot be attributed to a single agent. Direct causal links between pyrethroid exposure and SBS have not been established.

In summary, pyrethroids' physicochemical persistence, interaction with dust and carpets, and recirculation through HVAC systems create a chronic exposure environment in which residues persist long after spraying. From this perspective, an indoor pyrethroid exposure pathway (exposome) framework may help to link exposure dynamics with biological mechanisms of long-term toxicity and to support clinical surveillance and prevention recommendations while evidence-based guidance is being developed.

## 4 The indoor pyrethroid exposure pathway: carpets, dust reservoirs, resuspension, and HVAC redistribution

Indoor environments profoundly influence the persistence and movement of pyrethroid residues. Unlike outdoor applications, where sunlight, rainfall, and air exchange promote degradation, enclosed workplaces provide minimal dilution and photodegradation potential (3, 8). The hydrophobic nature of pyrethroids favors their adsorption to dust particles, fibers, and porous surfaces such as carpets and upholstered furniture (8, 9). Over time, these materials act as **reservoirs**, storing residues that can later re-enter the breathing zone or contact surfaces through resuspension.

Carpeted flooring plays a particularly significant role in this process. Wall-to-wall carpets possess large surface areas and complex fiber structures that trap fine particulate matter and semi-volatile compounds (3, 8, 9). Residues accumulate within the dust matrix and persist for weeks or months, depending on compound type, cleaning frequency, and ambient temperature. Mechanical disturbance from walking, vacuuming, or air flow readily resuspends contaminated dust, maintaining background concentrations that are decoupled from recent spraying events. Repeated applications, especially in poorly ventilated or heavily carpeted areas, therefore sustain a chronic exposure cycle even when spraying frequency appears moderate (8, 9, 26).

Central heating, ventilation, and air-conditioning (HVAC) systems add a further dimension to exposure dynamics. During operation, HVAC units can draw dust from contaminated zones, redistribute particles through ducts, and deposit residues on filters and interior surfaces (10, 28). Partial recirculation of air increases the potential for redistribution rather than elimination of contaminants. If maintenance is infrequent or filters are inefficient, accumulated residues may be released during subsequent air-handling cycles. This mechanism extends exposure both spatially (to untreated rooms) and temporally (long after initial spraying). Few studies have quantitatively assessed HVAC-mediated redistribution, representing an evidence gap in chronic indoor exposure assessment (10, 26).

Resuspension and redistribution together produce a **“**dust-reservoir-driven**”** exposure loop that may challenge the conventional view of pesticide safety based solely on immediate post-spray measurements. Occupants in such settings experience repeated low-level exposure through inhalation of particle-bound residues, dermal contact with contaminated surfaces, and unintentional ingestion via hand-to-mouth activity.

Existing occupational guidelines largely ignore these pathways, focusing instead on direct spray contact and acute toxicity (23, 26). Integrating dust-reservoir dynamics and HVAC behavior into risk-assessment models may be essential for accurate estimation of chronic indoor exposure and for the design of effective prevention strategies.

### 4.1 Occupational indoor pyrethroid exposure pathway framework

**Figure 1:**
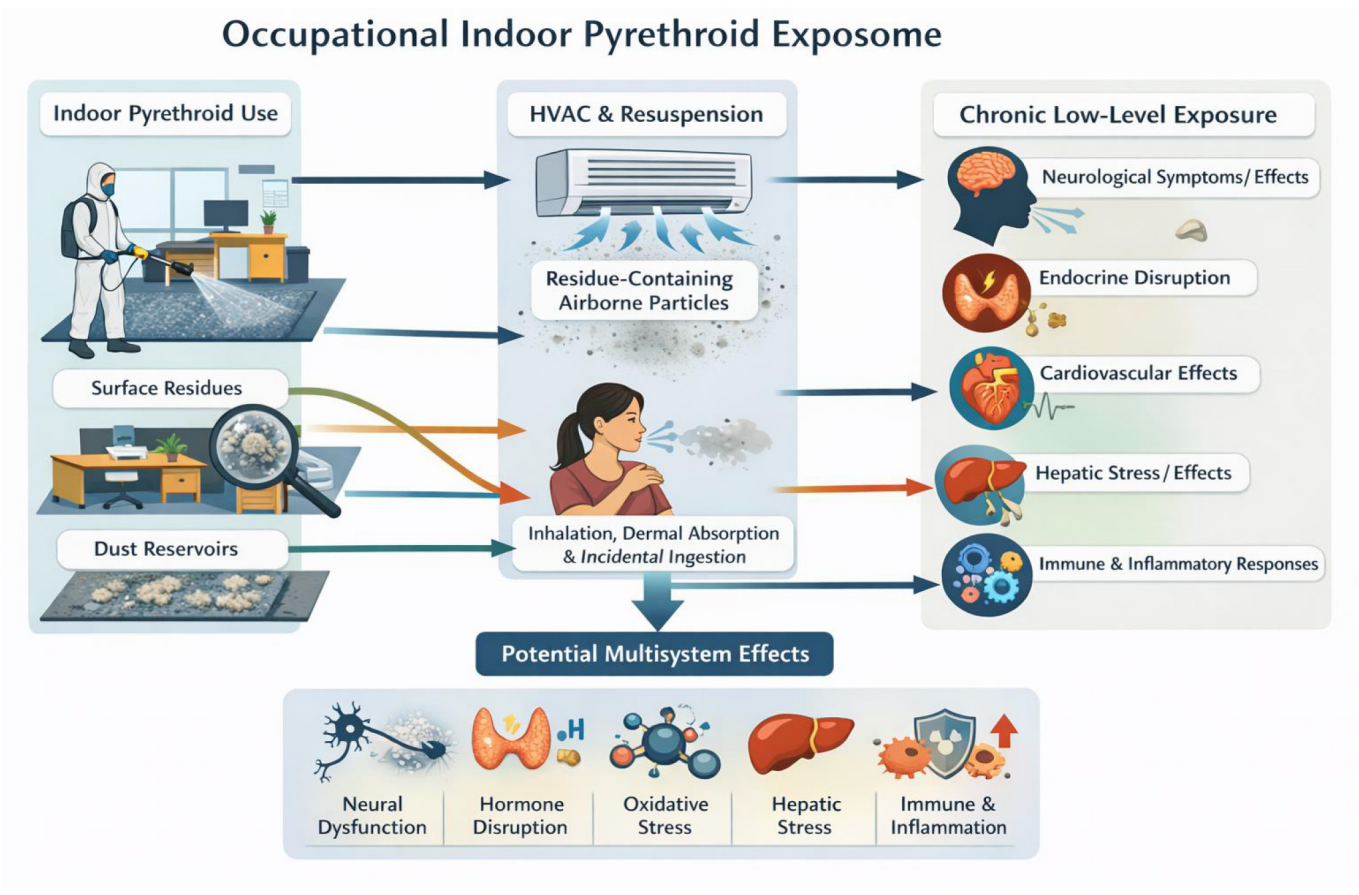
Occupational indoor pyrethroid exposure pathway framework.

Schematic representation of the occupational indoor pyrethroid long-term exposure (Figure 1). Indoor spraying leads to residue accumulation and chronic low-level exposure via resuspension and HVAC redistribution, with associated chronic health effects. Original figure created by the first author AAMK.

## 5 Mechanistic pathways of chronic toxicity

This section summarizes mechanistic and experimental evidence.

Pyrethroids exert toxicity primarily through interference with neuronal ion channels, but chronic low-dose exposure engages a broader range of biological mechanisms that extend beyond acute neuroexcitation. These mechanisms collectively explain the multisystem effects reported in experimental and human studies (14, 15, 24, 30).

### 5.1 Neurotoxicity and mitochondrial dysfunction

Both Type I and Type II pyrethroids act on voltage-gated sodium channels, delaying their inactivation and causing repetitive neuronal firing (4, 5). Type II compounds such as lambda-cyhalothrin also affect calcium and chloride channels, further disturbing membrane potential. With repeated low-level exposure, secondary effects emerge: oxidative stress, mitochondrial membrane depolarization, and impaired ATP production (14–16). These changes contribute to neuronal fatigue, altered neurotransmitter metabolism, and cognitive or mood disturbances consistent with chronic exposure symptoms observed in workers (19, 25, 31).

In experimental models, persistent mitochondrial dysfunction and reactive oxygen species (ROS) generation link pyrethroid exposure to neurodegeneration-like outcomes (14–16, 20).

### 5.2 Hepatotoxicity

The liver is a major site of pyrethroid biotransformation through cytochrome P450-mediated oxidation and ester hydrolysis. Prolonged exposure can induce hepatic enzyme activity, increasing serum ALT, AST, and γ-glutamyl transferase (GGT) levels (15, 32, 33). Studies in animals show hepatocellular hypertrophy and oxidative injury following chronic low-dose intake of lambda-cyhalothrin or cypermethrin (15, 16). Mechanistically, hepatotoxicity arises from mitochondrial ROS generation, lipid peroxidation, and depletion of antioxidant defenses, leading to metabolic stress and potential progression toward steatohepatitis-like pathology (15, 16, 32). These findings are consistent with occupational reports of elevated liver enzymes among chronically exposed workers.

### 5.3 Endocrine and thyroid disruption

Pyrethroids can alter endocrine homeostasis by interfering with thyroid hormone synthesis, transport, and metabolism. Several studies demonstrate inverse or nonlinear associations between urinary pyrethroid metabolites and circulating T3/T4 levels in adults (17, 18, 22). Proposed mechanisms include oxidative inhibition of thyroid peroxidase, disruption of deiodinase enzymes, and competitive binding of pyrethroid metabolites to transthyretin, the thyroid hormone transport protein (21). Chronic oxidative burden may further exacerbate thyroid vulnerability, particularly in individuals with pre-existing endocrine or autoimmune disorders (17, 18, 21, 22, 24).

### 5.4 Cardiovascular and vascular effects

Epidemiological findings suggest associations between chronic pyrethroid exposure and increased cardiovascular risk, including hypertension and endothelial dysfunction (19, 26). Mechanistically, vascular effects may stem from oxidative stress–induced nitric oxide depletion, mitochondrial impairment in vascular smooth muscle, and autonomic imbalance secondary to neuronal hyperexcitability (19, 23, 26, 31). These processes may collectively contribute to altered heart-rate variability and inflammatory vascular responses observed in chronically exposed populations.

### 5.5 Immune and inflammatory pathways

Chronic low-dose exposure can shift immune balance toward a pro-inflammatory state, characterized by elevated cytokine production and activation of microglia or peripheral macrophages (14, 15, 20). Experimental studies indicate that pyrethroids can enhance oxidative and inflammatory signaling through NF-κB and MAP-kinase pathways. Persistent low-grade inflammation may contribute to fatigue, allergic-like symptoms, and immune dysregulation observed in some exposed workers (20, 24, 28, 29).

### 5.6 Summary

Combined activation of oxidative, inflammatory, endocrine, and mitochondrial pathways may provide a coherent explanation for multisystem toxicity observed with chronic indoor pyrethroid exposure which may contribute to cumulative hepatic, thyroid, neurological, cardiovascular, and immune dysfunctions despite the absence of overt acute poisoning. Mechanistic pathways and their biological targets are summarized in Table 1. Even some studies also suggest potential genotoxic effects, including DNA damage in human cells following pyrethroid exposure (34).

**Table 1:** Mechanistic pathways of chronic indoor pyrethroid toxicity and representative evidence.

Mechanistic pathway	Target system(s)	Key biological effects	Representative references
Oxidative stress	Nervous, hepatic, thyroid	Reactive oxygen species generation, lipid peroxidation, antioxidant depletion	(14–16, 21)
Mitochondrial dysfunction	Nervous, cardiovascular	Membrane depolarization, ATP depletion, altered neurotransmission	(15, 16, 20, 23)
Endocrine and thyroid disruption	Endocrine, thyroid	Altered T3/T4 and TSH levels, thyroid peroxidase inhibition, transthyretin binding	(17, 18, 21, 22, 24)
Inflammatory signaling	Immune, vascular	NF-κB and MAPK pathway activation, cytokine upregulation, chronic low-grade inflammation	(14, 15, 20, 24)
Vascular and autonomic imbalance	Cardiovascular	Nitric oxide depletion, endothelial dysfunction, heart–rate variability changes	(19, 23, 26, 31)
Hepatic enzyme induction	Hepatic, metabolic	Cytochrome P450 activation, elevated ALT/AST/GGT, hepatic oxidative injury	(15, 16, 24, 32, 33)

### 5.7 Mechanistic pathways of chronic indoor pyrethroid toxicity and representative evidence

Mechanistic pathways of chronic indoor pyrethroid toxicity and representative evidence are summarized in (Table 1). Original table created by the first author AAMK.

## 6 Human evidence: biomonitoring and epidemiological findings

This section presents human biomonitoring and epidemiological evidence.

### 6.1 Biomonitoring of indoor exposure

Urinary metabolites such as 3-phenoxybenzoic acid (3-PBA) and DCCA are commonly used to assess exposure to pyrethroid insecticides. Because pyrethroids are rapidly metabolized, these biomarkers reflect recent exposure, typically within the previous one to three days. Urinary biomarkers do not measure cumulative or long-term body burden and cannot quantify chronic exposure on their own (35, 36). Their main value is to indicate ongoing or repeated exposure when detected repeatedly over time. In routine occupational practice, urinary biomonitoring for pyrethroids is rarely performed, due to the lack of validated threshold values, limited clinical interpretability, and absence of regulatory requirements. As a result, assessment of long-term exposure usually relies on exposure history, workplace conditions, environmental persistence (e.g., dust and surface residues), and clinical findings, rather than urine testing alone.

Studies from many countries have found pyrethroid residues or metabolites in people who live or work indoors, even when they are not directly involved in spraying (1–3, 7). The main urinary markers-−3-phenoxybenzoic acid (3-PBA) and cis/trans-DCCA—show that exposure occurs through breathing contaminated air, contact with dust, and skin absorption. National surveys in the United States and Europe report that most adults and children have detectable levels of these metabolites (1, 2, 25). Concentrations are higher in people working or living in recently sprayed buildings, especially where ventilation is poor or carpets trap dust (3, 8, 9).

Workers in pest control, cleaning, and building maintenance jobs often have the highest levels. Their urine samples show repeated low-dose exposure far above background levels (2, 3, 26).

However, very few studies have tracked workers over time, and there are no clear reference values defining what levels are safe or harmful. Currently, no well-established threshold values exist for chronic indoor pyrethroid exposure. Biomonitoring markers such as 3-phenoxybenzoic acid (3-PBA) and DCCA primarily reflect recent exposure and do not provide clear thresholds for long-term risk assessment. This lack of long-term monitoring and validated exposure limits represents an important limitation in interpreting chronic exposure and highlights a major gap in protecting indoor workers (23, 26).

### 6.2 Nervous system effects

People with long-term exposure to pyrethroids often report headaches, dizziness, fatigue, or slower thinking (4, 5, 14–16, 19). Several studies link higher 3-PBA levels with poorer results on attention and coordination tests (19, 28, 31).

These effects are believed to result from oxidative stress and mitochondrial damage in nerve cells, which cause gradual loss of energy and nerve signaling problems (14–16, 20). Such changes fit the pattern seen in animal studies and explain the chronic fatigue and concentration problems reported by many exposed workers.

Epidemiological studies have also explored associations between pesticide exposure and neurological outcomes, including brain tumors, although findings remain inconsistent (24, 27, 30).

### 6.3 Thyroid and hormonal effects

Research from Asia and Europe shows that pyrethroid exposure can disturb thyroid hormone balance (17, 18, 22). Workers and residents exposed to these chemicals often have changes in blood levels of T3, T4, and TSH, suggesting that the thyroid is under stress (17, 18, 21, 22). Laboratory studies show that the metabolite 3-PBA can attach to transthyretin, a protein that normally carries thyroid hormones in the blood, possibly reducing hormone availability (21). Despite this evidence, there are still no official limits for long-term exposure or guidance on safe spraying intervals (23, 26).

### 6.4 Liver and metabolic effects

In some occupational settings, mild but persistent elevations of liver enzymes, particularly GGT and ALT, have been observed in exposed workers despite the absence of common alternative causes. Viral hepatitis markers are negative, abdominal ultrasound findings are normal, and there is no history of alcohol use, metabolic liver disease, or other identifiable hepatotoxic exposures. In this context, chronic low-dose chemical exposure represents a plausible contributing factor to subclinical hepatic stress. Experimental and occupational studies have shown that pyrethroids can induce hepatic enzyme activity through oxidative stress and mitochondrial dysfunction, supporting a biologically plausible link. However, these findings remain associative rather than diagnostic and should be interpreted within a comprehensive clinical and occupational assessment (15, 32, 33).

Laboratory studies support this, showing that repeated low-dose exposure can trigger oxidative stress and enzyme induction in liver tissue (15, 16). These findings are consistent with chronic exposure in indoor workplaces.

### 6.5 Heart and circulation

A long-term U.S. population study found that adults with higher urinary pyrethroid levels had a greater risk of heart disease and early death (23, 26).

Other studies suggest that chronic exposure may damage the inner lining of blood vessels, affect heart-rate control, and increase inflammation (19, 23, 26). Such effects point to broader systemic toxicity that current short-term safety guidelines fail to consider (23, 26).

### 6.6 Immune and irritation symptoms

People working in heavily sprayed or poorly ventilated buildings often report tiredness, throat and eye irritation, cough, or allergy-like reactions (24, 28, 29). Some workers in enclosed workplaces report symptoms such as headache, fatigue, dizziness, mucosal irritation, or difficulty concentrating, which improve after leaving the building. This reversible symptom pattern is consistent with Sick Building Syndrome–type presentations, which are defined by nonspecific symptoms linked to time spent in a particular indoor environment. Sick Building Syndrome is multifactorial and cannot be attributed to a single agent (37). In settings with recurrent indoor pesticide use, residue persistence, and limited ventilation, chemical exposures may represent one of several contributing environmental factors, alongside ventilation quality, indoor air characteristics, and organizational conditions. Direct causal links between pyrethroids and SBS have not been established.

These complaints are consistent with studies showing that pyrethroids can activate inflammatory and immune pathways, raising levels of cytokines and oxidative markers (14, 15, 20). This ongoing low-grade inflammation may explain *Sick Building Syndrome*–like symptoms reported in chronically exposed workers. In addition, some studies have described transient neuro-sensory symptoms such as paresthesia (tingling or burning skin sensations), particularly involving the face, which are more characteristic of pyrethroid exposure. Despite these clues, immune and inflammatory effects are rarely included in workplace health monitoring.

## 7 Occupational risk management and medical surveillance agenda

### 7.1 Concept and purpose

Occupational exposure to pyrethroids in enclosed, air-conditioned workplaces may represent an under-recognized chronic health risk. Most safety frameworks address only acute toxicity and short-term re-entry precautions, although repeated indoor spraying creates a continuous low-dose exposure cycle that is not captured by current regulations (23, 26). This section proposes a structured framework for potential risk management and medical surveillance, designed to prevent or to reduce a potential risk of chronic toxicity and to support early detection of exposure-related health effects.

### 7.2 Exposure control and prevention strategies

Effective control begins with accurate exposure documentation and operational discipline. Each spraying event should be recorded, including product identity, active ingredient, concentration, and treated zones. Ventilation and HVAC systems require active management—shutdown during application and controlled restart only after sufficient air exchange (10, 28). Whenever possible, Integrated Pest Management (IPM) should replace routine calendar-based spraying with pest-threshold – based justification to minimize unnecessary chemical use (3, 8, 9).

Post-application cleaning using HEPA-filtered vacuuming is essential in carpeted buildings to reduce dust-reservoir residues. Regular surface or dust sampling can be used to verify the effectiveness of these control measures and provide real exposure data for occupational-health teams (23, 26).

### 7.3 Worker health protection and surveillance

This review primarily focuses on workers or employees who occupy indoor environments for prolonged periods following pesticide application. It may also consider individuals directly involved in pesticide application in enclosed workplaces in the long term.

Chronic indoor exposure to pyrethroids may affect several biological systems, including the liver, thyroid, nervous system, cardiovascular, and immune functions (14–22, 26, 32). Therefore, worker health programs may consider combining routine medical screening, symptom-based evaluation, and targeted follow-up.

Annual medical assessments also may consider including a structured symptom questionnaire covering fatigue, dizziness, headaches, eye or throat irritation, skin changes, and other exposure-related complaints. The physical examination should record vital signs, blood pressure, and body mass index. Laboratory testing may consider focusing on affordable and reliable markers of early biological stress. These may include liver function tests (ALT, AST, and GGT) to detect hepatic enzyme induction (15, 24, 32, 33), thyroid function tests (TSH and free T4) to identify endocrine disruption (17, 18, 21, 22, 24), and a metabolic profile (fasting glucose, HbA1c, and lipid panel) to assess oxidative or metabolic stress (20). Renal and hematologic parameters such as serum creatinine, estimated glomerular filtration rate, and complete blood count can be also evaluated to monitor general health status (23).

These assessments can be performed annually as part of routine occupational health screening and may identify early organ stress when direct exposure data are unavailable. Additional investigations should be guided by symptoms, such as spirometry for persistent respiratory complaints, electrocardiography for cardiovascular symptoms, or neurological evaluation when indicated.

Urinary metabolite testing (e.g., 3-PBA or DCCA) may be used selectively in unexplained cases or research settings but is not routinely feasible and should complement, not replace, clinical assessment (1–3, 23).

Vulnerable workers, including those with asthma, thyroid disease, pregnancy, or autoimmune conditions, may require individualized exposure reduction measures such as task modification or relocation. Follow-up medical evaluations should be documented and coordinated with environmental records.

This integrated surveillance approach links occupational hygiene, ventilation control, and medical monitoring to support early detection of exposure-related effects and reduce long-term health risks.

### 7.4 Implementation framework

To support consistent application, Table 2 presents a structured occupational checklist integrating exposure documentation, operational controls, environmental monitoring, and medical surveillance. It is potentially intended as a practical template for workplaces where pyrethroids are used regularly and formal safety standards may be lacking. This checklist translates the exposure pathway framework concept into routine occupational practice, linking record-keeping, ventilation management, and worker health monitoring in one framework.

**Table 2:** Occupational safety, exposure control, and medical surveillance checklist for recurrent indoor pyrethroid spraying environments.

Category	Key component	Specific requirements & actions	Documentation/Evidence
**Exposure documentation & operational controls**	Chemical & Process Records	Maintain SDS/MSDS, records of active ingredients, application dates, treated zones, and spraying frequency.	Binder/Electronic Log: “Spray Log & SDS”
**Exposure documentation & operational controls**	Standard Operating Procedures (SOPs)	Implement written SOPs for pre-spray notification, area evacuation, re-entry time criteria, and HVAC control.	Posted SOPs: “Notification/Evacuation/Re-entry”
**Exposure documentation & operational controls**	Ventilation Management	Document HVAC shutdown/restart timing and post-application ventilation duration.	Log Entry: “HVAC Shutdown/Restart Log”
**Exposure documentation & operational controls**	Spray Justification	Justify spraying frequency using Integrated Pest Management (IPM) principles to minimize unnecessary use.	IPM Log: “Pest Sighting & Action Threshold Record”
**Exposure documentation & operational controls**	Post-Application Cleaning	Ensure cleaning strategies (e.g., HEPA vacuuming) are appropriate for carpeted environments to reduce residue.	SOP: “Post-Spray Cleaning for Carpets”
**Exposure documentation & operational controls**	Health Reporting	Maintain a structured system for workers to report symptoms or incidents related to spraying.	Form: “Employee Symptom/Incident Report”
**Environmental monitoring (recommended)**	Residue Sampling	Conduct periodic surface wipe and/or dust sampling, especially in carpeted areas, to estimate residue persistence.	Report: “Quarterly Surface Wipe Sampling Results”
**Routine medical surveillance (annual minimum)**	Clinical Assessment	Structured Symptom Questionnaire: SBS-pattern, neuro-sensory (tingling, dizziness), cardiovascular (palpitations). Physical: Blood pressure, BMI. Blood Panel: CBC, Liver Enzymes (ALT/AST/GGT), Kidney (Creatinine/eGFR), Metabolic (Fasting Glucose/HbA1c, Lipids), Thyroid (TSH, Free T4).	Form/Report: “Annual Occupational Health Screening”
**Symptom-triggered add-on testing**	Respiratory	Spirometry for workers with persistent cough, wheezing, or shortness of breath.	Referral/Report: “Pulmonary Function Test”
**Symptom-triggered add-on testing**	Cardiovascular	ECG for workers with persistent palpitations, chest discomfort, or hypertension.	Referral/Report: “Electrocardiogram Report”
**Symptom-triggered add-on testing**	Neurological	Clinical neurological evaluation; consider nerve conduction studies if peripheral neuropathy is suspected.	Referral/Report: “Neurology Consultation”
**Biomonitoring (optional)**	Internal Dose Assessment	Measure urinary pyrethroid metabolites (e.g., 3-PBA, DCCA) to support exposure assessment, especially when paired with environmental data.	Report: “Urinary Metabolite Analysis Series”
**Worker protection & accommodation**	Vulnerable Worker Management	Identify vulnerable workers (e.g., with asthma, skin conditions, pregnancy). Implement exposure reduction strategies: task modification, relocation, enhanced PPE, remote work.	Plan: “Individual Exposure Control Plan”
**Worker protection & accommodation**	Follow–Up	Conduct follow–up health monitoring for any worker with recurrent symptoms potentially linked to exposure.	Record: “Accommodation & Follow-Up Log”

### 7.5 Occupational safety, exposure control, and medical surveillance checklist for recurrent indoor pyrethroid spraying environments

A structured occupational safety, exposure control, and medical surveillance checklist for recurrent indoor pyrethroid spraying environments is presented in (Table 2). Original table created by the first author AAMK.

## 8 Discussion and recommendations

### 8.1 Interpretation of findings

This review indicates that chronic low-level exposure to pyrethroids can occur in enclosed workplaces, mainly through residue persistence in dust and carpets and repeated resuspension via HVAC systems (3, 8–10). Because pyrethroids readily bind to indoor surfaces, workers may experience ongoing background exposure long after spraying.

Experimental and human studies suggest that long-term exposure may be associated with gradual effects across several systems, including the nervous, liver, endocrine, cardiovascular, and immune systems (4, 5, 14–23, 26, 32). Shared mechanisms such as oxidative stress, mitochondrial dysfunction, and endocrine disruption help explain these findings.

Current safety frameworks focus on acute exposure and short-term re-entry and do not address cumulative indoor exposure. As a result, guidance on spraying frequency, ventilation duration, and health monitoring in enclosed buildings remains limited (23, 26). While some studies report minimal effects under well-controlled conditions, variability in formulation, ventilation, and application practices highlights the need for further long-term, exposure-controlled studies.

### 8.2 Strengths and practical implications

This review brings together mechanistic toxicology, exposure science, and occupational medicine to frame indoor pesticide use as a chronic exposure issue. It provides a practical basis for workplace safety by linking environmental controls, medical surveillance, and occupational guidance. The occupational checklist (Table 2) supports documentation, exposure prevention, and health monitoring using feasible measures such as ventilation management, symptom tracking, and basic laboratory tests, including liver enzymes and thyroid function (15, 17, 18, 21, 22, 32).

### 8.3 Research priorities and occupational guidance needs

Future research should quantify residue persistence and resuspension under different building conditions to define safe re- entry and re-spraying intervals and clarify cumulative exposure–response relationships (1–3, 26). Prospective cohort studies among indoor workers are needed to assess long-term health outcomes and identify early indicators of thyroid, liver, cardiovascular and other systemic effects. Occupational pesticide safety guidance should better address chronic indoor exposure by incorporating residue thresholds, HVAC maintenance considerations, medical surveillance, and routine documentation of spraying practices. Although quantitative exposure data from HVAC-mediated redistribution are limited, available environmental studies suggest that indoor dust and air circulation systems may contribute to continuous low-level exposure, including in vulnerable populations such as children, individuals with diabetes, or those with other chronic neurological conditions.

### 8.4 Limitations

Assessing chronic exposure is difficult because pyrethroids are rapidly metabolized and most biomonitoring reflects only recent exposure. Evidence is limited by heterogeneous study designs, few longitudinal occupational studies, and inconsistent environmental measurements (25, 37). Mechanistic findings are largely based on animal or *in vitro* studies and require confirmation in human settings. Despite these limitations, available evidence supports an association with multisystem effects from chronic low-dose exposure (38–40).

Clinical and occupational recommendations presented in this review are exploratory and based on indirect and heterogeneous evidence.

## 9 Conclusions

Recurrent indoor pyrethroid spraying in enclosed workplaces can sustain chronic low-dose exposure through residue persistence in dust and textiles and HVAC-mediated redistribution, and may contribute to unrecognized chronic multisystem effects. Clinicians and occupational health practitioners may consider this exposure scenario when evaluating workers presenting with chronic fatigue, headache, dizziness, neurocognitive or mood symptoms, and subtle laboratory abnormalities such as mild elevated liver enzymes or thyroid dysfunction. A structured clinical approach incorporating targeted exposure history, symptom pattern recognition, and periodic surveillance (e.g., liver enzymes and thyroid function) may be considered as a precautionary approach in the absence of definitive evidence and may support early identification of potentially exposure-related illness. Importantly, the current lack of evidence-based guidance on indoor re-application frequency, re-application intervals, and ventilation-based safety measures, may represent an important occupational health guidance gap. Current regulatory frameworks primarily address acute exposure and do not adequately consider chronic indoor exposure pathways such as residue persistence and HVAC redistribution. Clinical surveillance and workplace prevention recommendations are needed until well-designed studies define safe indoor spraying practices and clear guidance for enclosed environments.
